# Sellar Schwannoma Masquerading as Giant Pituitary Adenoma: A Diagnostic Challenge

**DOI:** 10.1155/2024/6230715

**Published:** 2024-10-16

**Authors:** Ali Alkhaibary, Norah Mohammad Alotaibi, Ghaida Abdullah Albattah, Rahaf Alotaibi, Fahd AlSufiani, Ahmed Aloraidi

**Affiliations:** ^1^College of Medicine, King Saud bin Abdulaziz University for Health Sciences, Riyadh, Saudi Arabia; ^2^King Abdullah International Medical Research Center, Riyadh, Saudi Arabia; ^3^Division of Neurosurgery, Department of Surgery, King Abdulaziz Medical City, Ministry of National Guard—Health Affairs, Riyadh, Saudi Arabia; ^4^Department of Neurosurgery, National Neurosciences Institute, King Fahad Medical City, Riyadh, Saudi Arabia; ^5^Department of Pathology and Laboratory Medicine, King Abdulaziz Medical City, Ministry of National Guard—Health Affairs, Riyadh, Saudi Arabia

**Keywords:** parasellar, schwannoma, sella

## Abstract

**Background:** Schwannomas are well-encapsulated, solitary tumors that grow slowly from the nerve sheath. Sellar schwannomas tend to be mistaken for other sellar/parasellar lesions due to similar clinical and radiological findings. The present article describes the clinical presentation, radiological findings, histopathological features, and outcome of a patient with sellar schwannoma.

**Case Description:** A 23-year-old female, known to have hypothyroidism secondary to Hashimoto's thyroiditis, presented with multiple episodes of galactorrhea, weight gain, and irregular menstrual cycle for 8 months. It was associated with decreased visual acuity and episodic headaches. Neurological examination revealed no focal deficits. Brain magnetic resonance imaging (MRI) showed a well-defined lobulated lesion in the sellar region, compressing the right optic nerve and optic chiasm. The patient underwent craniotomy and tumor resection. The histopathological sections were diagnostic of schwannoma. Postoperatively, the patient noted a subjective improvement in her visual acuity. She was discharged in stable condition with regular follow-ups at neurosurgery, endocrine, and ophthalmology clinics.

**Conclusion:** Schwannoma of the sellar region is rare and can be misdiagnosed as pituitary adenomas. Preoperative hormonal profile and meticulous neuroradiological assessment narrow down the differential diagnosis for patients with sellar lesions. The diagnosis of sellar schwannomas is established with histopathology and immunohistochemistry results.

## 1. Introduction

Schwannomas, which are also called neurinomas or neurilemmomas, are well-encapsulated, solitary tumors that grow slowly from the nerve sheath. They are composed of Schwann cells that originate from the neural crest. The World Health Organization (WHO) designates schwannoma as a grade I benign tumor. Though usually solitary, multiple tumors may indicate neurofibromatosis type 2, schwannomatosis, or Carney complex [1]. Schwannomas represent approximately 89% of nerve sheath tumors, with vestibular schwannomas comprising 57% of benign schwannomas [2].

Sellar schwannomas tend to be mistaken for other sellar/parasellar lesions due to similar clinical and radiological findings. A comprehensive literature review reveals only 25 cases of sellar schwannomas. The present article describes the clinical presentation, radiological findings, histopathological features, and outcome of a patient with sellar schwannoma.

## 2. Case Description

### 2.1. History

A 23-year-old female, known to have hypothyroidism secondary to Hashimoto's thyroiditis, presented with multiple episodes of galactorrhea, weight gain, and irregular menstrual cycle for 8 months. It was associated with decreased visual acuity and episodic headache.

### 2.2. Physical Examination

Neurological examination revealed no focal deficits. The visual acuity was 20/25 on the right side and 20/20 on the left side. No visual field defects nor papilledema were noted. The cranial nerve examination was grossly intact.

### 2.3. Neuroradiological Imaging

**Figure 1:**
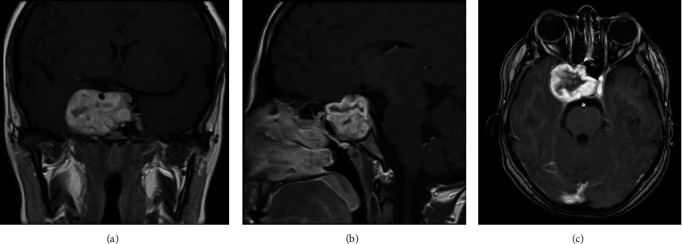
(A–C) Coronal, sagittal, and axial brain MRI with contrast showing a well-defined heterogeneously enhancing lesion in the sellar region measuring about 4.5 cm × 4 cm × 4 cm. It is displacing the medial surface of the right temporal lobe and compressing on the right optic nerve and optic chiasm. The right internal carotid artery is completely encased by the lesion and displaced superiorly. MRI, magnetic resonance imaging.

Brain magnetic resonance imaging (MRI) showed a well-defined lobulated lesion in the sellar region, compressing the right optic nerve and optic chiasm (Figure 1).

### 2.4. Surgical Intervention

Considering the progressive deterioration in visual acuity and the large sellar component of the tumor, the patient underwent craniotomy and tumor resection. Intraoperatively, some of the tumor tissues were situated deep to the roots of the trigeminal nerve. Therefore, the tumor was visualized and dissected meticulously in piecemeal, preserving the normal parenchyma. The tumor was resected and sent to frozen and permanent pathology.

### 2.5. Histopathological Findings

**Figure 2:**
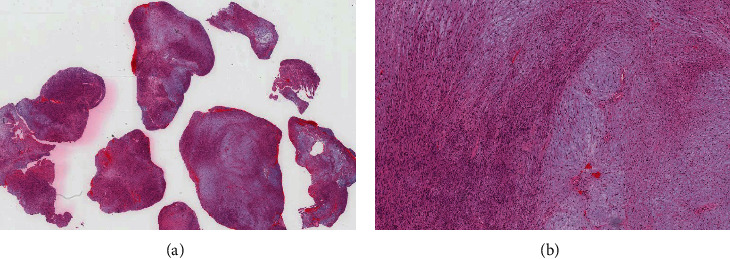
(A) H&E-stained section reveals multiple fragments of lesional tissue with variable cellularity and areas of hemorrhage. (B) Medium-power view showing hypercellular Antoni A “left side” and hypocellular Antoni B “right side” areas.

**Figure 3:**
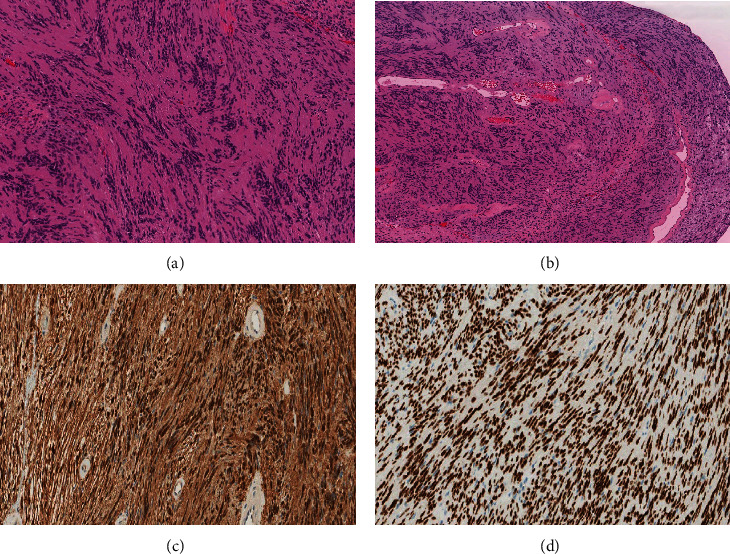
(A) High-power view of the spindle cells in Antoni A zones of schwannoma that typically show indistinct cytoplasmic borders and elongated, buckled, wavy nuclei embedded in fine, fibrillary, and eosinophilic matrix with characteristic nuclear palisading. (B) A characteristic finding in schwannoma is the presence of single or clusters of irregular vascular channels with hyalinized walls. (C and D) S100 and SOX-10 immunohistochemical stains are diffusely and strongly positive in schwannoma.

Examination of hematoxylin and eosin (H&E)-stained slides revealed multiple fragments of lesional tissue composed of spindle cells arranged in fascicles with palisading and intervening hyalinized blood vessels. The spindle cells were monotonous, with wavy nuclei and inconspicuous nucleoli. Scattered cells with degenerative nuclear atypia were noted. Myxoid degeneration and cystic changes were present. Chronic inflammatory cells were identified. No atypia or increased mitotic activity was noted. S100 and SRY related HMG box 10 protein (SOX)-10 immunohistochemical stains were diffusely and strongly positive. The findings were consistent with Schwannoma (central nervous system [CNS] world health organization [WHO] grade 1) (Figures 2 and 3).

### 2.6. Outcome and Follow-Up

Postoperatively, the patient noted a subjective improvement in her visual acuity. The patient complained of mild numbness on the right side of V1–V3 distribution, which has resolved gradually over the days. She was discharged in stable condition with regular follow-ups at neurosurgery, endocrine, and ophthalmology clinics.

## 3. Discussion

The clinical presentation of nonpituitary sellar lesions is dependent upon several factors, including size, growth pattern, and impact on pituitary function [3]. Distinguishing between pituitary adenomas and nonpituitary sellar lesions often poses a clinical challenge [3]. Clinical manifestations observed in individuals with sellar schwannomas commonly include visual disturbances, characterized by visual field defects and decreased visual acuity [4]. Additional symptoms include seizures, reduced libido, apoplexy, hydrocephalus, headaches, and painful ophthalmoplegia, depending on the mass effect exerted by the lesion [4].

MRI, both with and without contrast, is the preferred imaging modality for the evaluation of suspected sellar or parasellar pathologies [5]. On MRI, schwannomas typically exhibit isointensity to hypointensity on T1-weighted sequences and hyperintensity on T2-weighted sequences [5]. Avid contrast enhancement is typically noted [5]. The heterogeneity of signal intensity on T1/T2-weighted images of schwannomas varies based on the presence of cystic changes and, less frequently, the occurrence of hemorrhage or calcifications [6].

The definitive treatment for sellar schwannoma is surgical excision, to establish the diagnosis, particularly for atypical radiological findings or clinical manifestations [7]. A microscopic transsphenoidal approach or open craniotomy can be used [7]. For small lesions, a transsphenoidal approach is highly recommended as it allows good visualization and facilitates near-complete tumor removal with less morbidity compared to other approaches [7].

The utilization of preoperative MRI and computed tomography (CT) scans can predict surgical challenges from anatomical variations, vascular anomalies, or abnormal tumor features [8]. An accurate understanding of anatomy and multidisciplinary planning is essential for safely dealing with these complex surgeries [8].

The exact histopathogenesis of schwannomas within the sellar region is still unclear. Multiple theories have been postulated to explain such a phenomenon [7]. These include tumors originating from the lateral nerve plexus of the sella, perivascular Schwann cells of the medial wall of the sella, or sensory nerves of the dural reflections of the sellar region [7].

## 4. Conclusion

Schwannoma of the sellar region is rare and can be misdiagnosed as pituitary adenomas. Preoperative hormonal profile and meticulous neuroradiological assessment narrow down the differential diagnosis for patients with sellar lesions. The diagnosis of sellar schwannomas is established with histopathology and immunohistochemistry results.

## Data Availability

The data that support the findings of this study are available from the corresponding author upon reasonable request.
